# Activity of Fusion Prophenoloxidase-GFP and Its Potential Applications for Innate Immunity Study

**DOI:** 10.1371/journal.pone.0064106

**Published:** 2013-05-23

**Authors:** Bing Yang, Anrui Lu, Qin Peng, Qing-Zhi Ling, Erjun Ling

**Affiliations:** 1 Key Laboratory of Insect Developmental and Evolutionary Biology, Institute of Plant Physiology and Ecology, Shanghai Institutes for Biological Sciences, Chinese Academy of Sciences, Shanghai, People’s Republic of China; 2 Department of Applied Biology, Zhejiang Pharmaceutical College, Ningbo, People’s Republic of China; University of British Columbia, Canada

## Abstract

Insect prophenoloxidase (PPO) is essential for physiological functions such as melanization of invading pathogens, wound healing and cuticle sclerotization. The insect PPO activation pathway is well understood. However, it is not very clear how PPO is released from hemocytes and how PPO takes part in cellular immunity. To begin to assess this, three *Drosophila melanogaster* PPO genes were separately fused with GFP at the C-terminus (rPPO-GFP) and were over-expressed in S2 cells. The results of staining and morphological observation show that rPPO-GFP expressed in S2 cells has green fluorescence and enzyme activity if Cu^2+^ was added during transfection. Each rPPO-GFP has similar properties as the corresponding rPPO. However, cells with rPPO-GFP over-expressed are easier to trace without PO activation and staining. Further experiments show that rPPO1-GFP is cleaved and activated by *Drosophila* serine protease, and rPPO1-GFP binds to *Micrococcus luteus* and *Beauveria bassiana* spores as silkworm plasma PPO. The above research indicates that the GFP-tag has no influence on the fusion enzyme activation and PPO-involved innate immunity action *in vitro*. Thus, rPPO-GFP may be a convenient tool for innate immunity study in the future if it can be expressed *in vivo*.

## Introduction

Insect prophenoloxidase (PPO) has been studied for over a century [1]. PPO is a copper-containing enzyme that oxidizes mono-phenols to produce melanins [1], [2]. This family of proteins exist in microbes, plants, invertebrate and vertebrate animals [3]–[7], and each of them has some specific functions. Insect PPO is a very important innate immunity protein that can induce melanization of microbes and parasites thereby quickly and effectively eliminating them [1], [3], [8]–[10]. In addition to inducing melanization, insect PPO is also involved in wound healing by inducing hemolymph to clot [3], [11], [12]. In the hemocoel, PPO is produced by oenocytoids in circulation and in the hematopoietic organs [13]. PPO is also found in other types of hemocytes [13], [14], and in *Manduca sexta*, PPO can bind to the cellular surfaces of some hemocytes [15]. Hemocytes are normally considered as the only source of PPO in insects. However, epithelial cells in the silkworm hindgut were found to produce and release PPO into the hindgut lumen, where the activated PO is responsible for feces melanization and microbe flora regulation [16]. PPO was also found in the wing discs of silkworm. Since there are physical tubes that connect the wing disc to an attached hematopoietic organ, hemocytes were concluded to enter the wing disc and mistakenly release PPO inside the wing disc [17]. PPO was also identified in the hind wing of *Tribolium castaneum* [18]. However, it is unclear whether there is any physiological function for PPO in the wing discs or wings.

Insect PPO is an important component of humoral and cellular immunity [3], [19]–[21]. Based on the pioneering studies by several research teams [3], [19]–[21], the biochemical pathways leading to PPO activation and melanization are well understood. The zymogen PPO must be cleaved at a conserved site by terminal serine proteases, and this occurs upon the recognition of polysaccharides on the surface of microorganisms by pattern recognition proteins such as PGRP, βGRP and C-type lectins [3], [19]–[21]. Moreover, the terminal serine proteases also need cleaving to become activated, and serine protease inhibitors (serpins) negatively regulate this process [22], [23]. For example, in *M. sexta*, two terminal serine proteases, PAP2 and PAP3, become activated to cleave plasma PPO with the help of serine protease homologues (SPH), and this is negatively regulated by serpins [19], [24]. Besides inducing melanization on the surface of invading pathogens [8], [25], PPO is also responsible for melanization at wounds to prevent further infection [26]. To important disease vectors, PPOs of mosquitoes have been extensively studied and shown to be involved in the anti- *Plasmodium* and anti-bacterial response in both the midgut and the hemocoel [25], [27]–[31]. *Drosophila melanogaster*, as a powerful genetic tool, is an invaluable model for studying PPO activation and regulation [32]. Thus, work done in small dipteran insects has greatly increased our understanding of PPO activation [27], [28], [32].

Recently, discoveries of insect PPO function have expanded our knowledge on the regulation of the PPO pathway. Originally, PPO activation was thought to be an independent pathway. However, work on *Tenebrio molitor* shows that there is crosstalk between PPO activation and the Toll pathway [33], which presumably allows the insect to respond to infection more rapidly and effectively. *Drosophila* PPO was recently identified as an important component of the clotting system, and to be responsible for sclerotization and melanization around wounds [12], [34]. *Drosophila* has three PPO genes, PPO1 (CG5779), PPO2 (CG8193) and PPO3 (CG2952). PPO1 and PPO2 are produced by crystal cells [35], [36]. However PPO3 can be expressed in lamellocytes when *Drosophila* is infected by parasites [37]. In each species of mosquito, there is up to 10 PPO genes [8]–[10], [28], but there are normally 2–3 PPO genes in other species of insects [1], [3], [21]. We have no idea why mosquito needs so many PPO genes. Although PPO is a very important factor to induce the melanization of malaria parasites, we know very little of the role of each PPO in the process of melanization. Therefore, it is very important to identify their functions separately in the mosquito which might be helpful to fight against malaria transmission by mosquito. For this purpose, using *Drosophila* PPO genes as a model, the PPOs were recently over-expressed in eukaryotic and prokaryotic cells for identifying their biochemical properties [36], [38]. An important finding is that *Drosophila* PPO can be expressed even if there is not enough Cu^2+^ in the culture medium. The apo-rPPO (inactive) becomes holo-PPO (active) in the presence of Cu^2+^, which makes it convenient to express enough rPPO for purification [36], [38]. When the three rPPOs were over-expressed in S2 cells respectively, rPPO1 and rPPO2 needed additional Cu^2+^ to achieve a status that permits activation for subsequent L-DOPA or dopamine staining. However, rPPO3 did not need additional Cu^2+^ to become active [36]. A very interesting phenomenon is that when additional Cu^2+^ or substrate was added to the cells, S2 cells with rPPO3 over-expressed became auto-melanized. No cleavage of rPPO3 was discovered [36]. When expressed in different tissues of transgenic *Drosophila*, PPO3 can also induce auto-melanization in those tissues [39]. Further research shows that specific amino acids around the active site pocket affect each rPPO activity by influencing the pocket size [40]. rPPO3 has the largest pocket, and thus, substrate can enter the enzyme to cause auto-melanization even without being cleaved by serine protease, which is a kind of enzyme activity leakage [40].

In the field of insect PPO study, many interesting questions remain unanswered [1]. For example, how is PPO released from hemocytes into plasma? Since PPO has no signal peptide, it is probably released from hemocytes after cell lysis [3], which is also under control by some proteins and chemicals [11], [41]. If PPO could be tagged with a fluorescent protein like GFP, this and other questions could be more clearly answered.

In this study, we independently expressed in S2 cells three *Drosophila* PPOs after fusing each of them with GFP at the C-terminus (rPPO-GFP). Our results show that each rPPO-GFP has similar properties as the corresponding unmodified rPPO. Furthermore, purified rPPO1-GFP can be cleaved and activated by *Drosophila* serine proteases to become an active PO. Just like silkworm plasma PPO, rPPO1-GFP can also bind microorganisms after being mixed with silkworm plasma. These data suggest that *in vivo* expression of rPPO-GFP could be used for the study of immune processes involving the phenoloxidase pathway.

## Results

### Activities of Fusion Prophenoloxidase-GFP Expressed in S2 Cells

Three *Drosophila melanogaster* prophenoloxidase (PPO) cDNAs with GFP fused at the C-terminal (rPPO-GFP) were sub-cloned in S2 cells and over-expressed. When rPPO1, rPPO2 and rPPO3 were over-expressed, they exhibited different biochemical properties [36]. If there is not enough Cu^2+^ in the culture medium, rPPO1 and rPPO2 have no activity. Some of the rPPO3 does have activity immediately after being expressed (Table 1). The enzyme activities and fluorescence properties of each rPPO and rPPO1-GFP, rPPO2-GFP and rPPO3-GFP were studied and compared.

**Table 1:** Summary of the biochemical properties of each rPPO-GFP and the corresponding rPPO.

		PPO1		PPO2		PPO3	
		rPPO1	rPPO1-GFP	rPPO2	rPPO2-GFP	rPPO3	rPPO3-GFP
Cell staining	+Cu^2+^	Yes	Yes	Yes	Yes	Yes	Yes
	−Cu^2+^	No	No	No	No	Yes (Some cells)	Yes (Some cells)
Auto-melainzation	+Cu^2+^	No	No	No	No	Yes	Yes
	−Cu^2+^	No	No	No	No	Yes	Yes
PO activity	+Cu^2+^	Higher	Lower	Equal	Equal	Equal	Equal
	−Cu^2+^	No	No	No	No	Yes	Yes
Protein expression	+Cu^2+^	Yes	Yes	Yes	Yes	Yes	Yes
	−Cu^2+^	Yes	Yes	Yes	Yes	Yes	Yes

**Figure 1:**
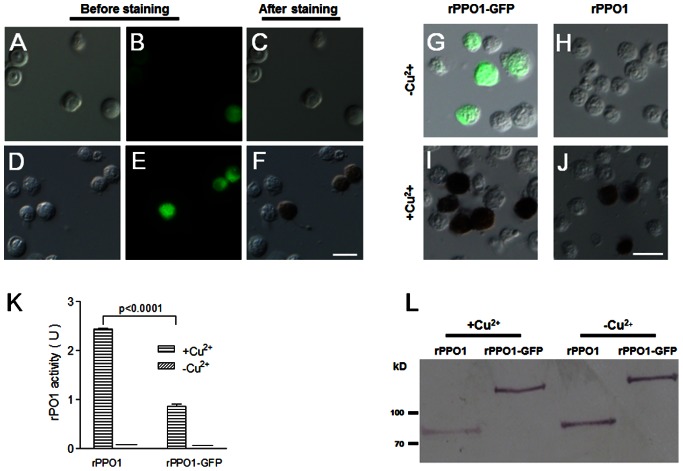
Phenoloxidase in S2 cells over-expressing rPPO1-GFP. DIC and fluorescence images were taken in the absence (A–C; −Cu^2+^) or presence (D–F; +Cu^2+^) of Cu^2+^ during cell transfection. In both cases, cells with green fluorescence were detected (A, B, D, E), and in the presence of Cu^2+^ those fluorescent cells displayed PO activity (dark brown) when incubated with dopamine dissolved in 30% ethanol (F). (G-J). Comparison of cells with rPPO1 and rPPO1-GFP expressed. rPPO1 and rPPO1-GFP were over-expressed in S2 cells in the absence (G, H) or presence (I, J) of Cu^2+^ during cell transfection. The cells were then stained for PO activity. Images represent DIC and fluorescence overlays. When Cu^2+^ was not added, no cells stained for PO activity (G, H) even though some cells expressed rPPO1-GFP (green fluorescence). When Cu^2+^ was added, many cells stained for PO activity (I, J; brown pigment), and no fluorescent cells were observed after PO staining due to quenching by melanin. (K) Comparison of rPPO1 and rPPO1-GFP enzyme activities. The amounts of rPPO1 and PPO1-GFP in S2 cell lysates were normalized and determined using purified rPPO1 as a standard by Western blot. Ethanol was used for enzyme activation. When Cu^2+^ was added, rPPO1 had significantly higher enzyme activity than rPPO1-GFP. No enzyme activities were detected if Cu^2+^ was not added, which is in agreement with the cell staining shown in (G-J). Columns represent the mean of individual measurements ± S.E.M (n = 3). Significant differences were calculated using the unpaired *t* test. (L) A Western blot showed that rPPO1-GFP and rPPO1 protein expression occurred regardless of the presence or absence of Cu^2+^. Bar: 20 µm.

rPPO1-GFP expressed in S2 cells was first identified by LC-MS/MS, and peptides from PPO1 and GFP were observed (Fig. S1, Table S2), which indicates that the fusion rPPO1-GFP can be expressed in S2 cells. When Cu^2+^ was not added during transfection, many cells had green fluorescence, indicating the expression of rPPO1-GFP (Fig. 1A–1C). However, no cells were stained, indicating no enzyme activity (Fig. 1C). If Cu^2+^ was added, S2 cells with GFP fluorescence became melanized after staining for enzyme activity (Fig. 1D–1E), indicating that rPPO1-GFP had enzyme activity after the addition of Cu^2+^ and activation by ethanol. Enzyme activities of rPPO1 and rPPO1-GFP were then compared. Without Cu^2+^ added during transfection, S2 cells with either rPPO1 or rPPO1-GFP did not stain for enzyme activity (Fig. 1G and 1H). If additional Cu^2+^ was provided, S2 cells with rPPO1 or rPPO1-GFP expressed were strongly stained for enzyme activity (Fig. 1I and 1J). These data corroborate that Cu^2+^ is necessary for apo-rPPO1 and apo-rPPO1-GFP to become holo-rPPO1 and holo-rPPO1-GFP. Finally, the enzyme activity of rPPO1-GFP was significantly lower than that of rPPO1 if Cu^2+^ was added (Fig. 1K), but the expression of rPPO1-GFP and rPPO1 was independent of Cu^2+^ addition during cell transfection (Fig. 1L).

**Figure 2:**
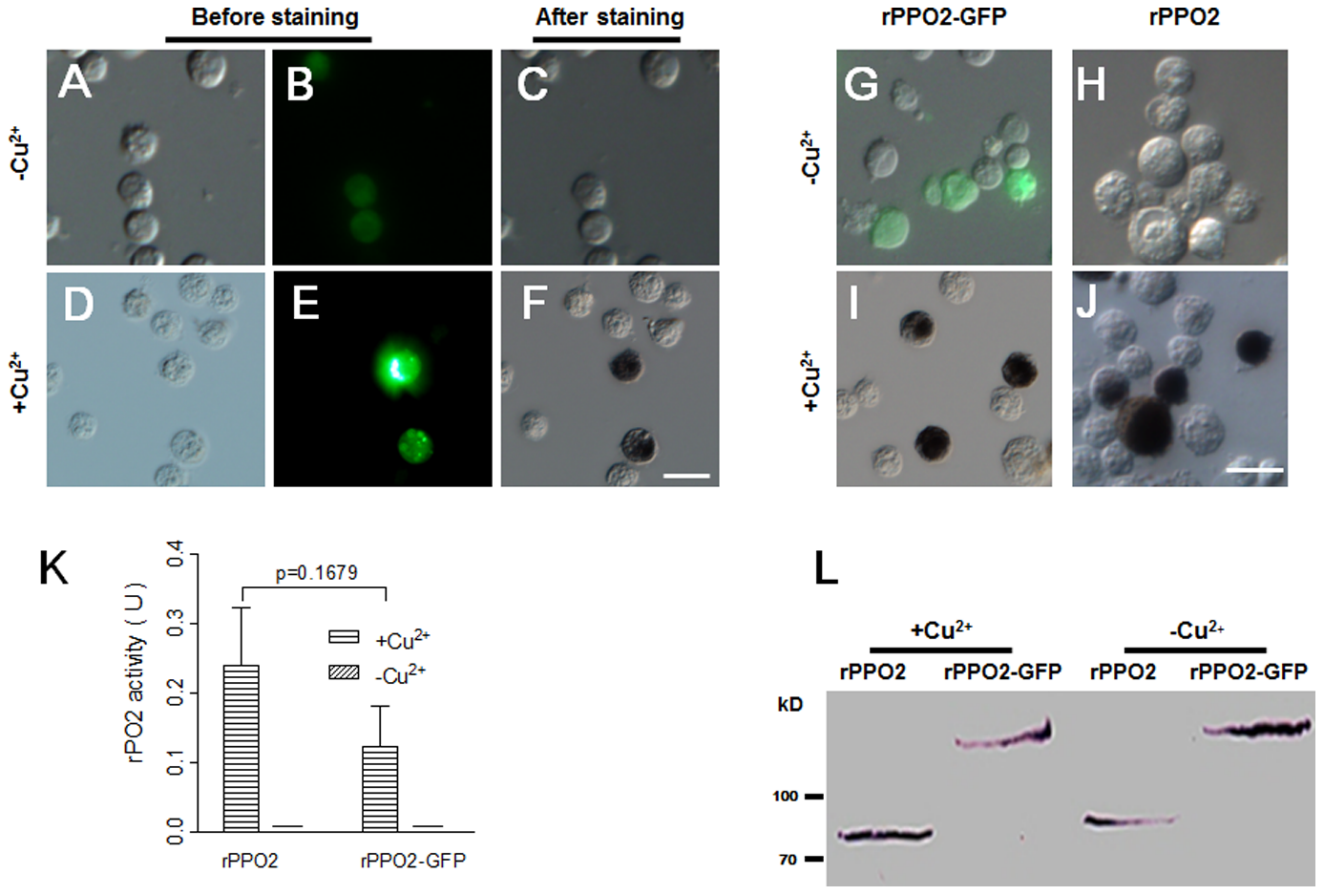
Phenoloxidase in S2 cells over-expressing rPPO2-GFP. DIC and fluorescence images were taken in the absence (A–C; −Cu^2+^) or presence (D–F; +Cu^2+^) of Cu^2+^ during cell transfection. In both cases, cells with green fluorescence were detected (A, B, D, E), and in the presence of Cu^2+^ those fluorescent cells displayed PO activity (dark brown) when incubated with dopamine dissolved in 30% ethanol (F). (G–J) rPPO2 and rPPO2-GFP were over-expressed in S2 cells in the absence (G, H) or presence (I, J) of Cu^2+^ for 48 h. When Cu^2+^ was not added, no cells stained for PO activity (G, H) even though some cells expressed rPPO2-GFP (green fluorescence). When Cu^2+^ was added, many cells stained to show PO activity (I, J; brown pigment), and no fluorescent cells were observed after PO staining due to quenching by melanin. (K) rPO2 activity assay as shown in Figure 1K. When Cu^2+^ was added, rPPO2 had the same enzyme activity as rPPO2-GFP. No enzyme activity was detected if Cu^2+^ was not added. Columns represent the mean of individual measurements ± S.E.M (n = 3). Significant differences were calculated using the unpaired *t* test. (L) Western blot showing that rPPO2-GFP and rPPO2 protein expression occurred regardless of the presence or absence of Cu^2+^. Bar: 20 µm.

For rPPO2-GFP, when Cu^2+^ was not added during transfection, S2 cells with green fluorescence did not stain for enzyme activity (Fig. 2A–2C), but they were positively stained if Cu^2+^ was added (Fig. 2D–2F). To compare rPPO2-GFP and rPPO2, fluorescence assays showed that, in the absence of Cu^2+^, only rPPO2-GFP had green fluorescence. But when Cu^2+^ was added, both rPPO2-GFP and rPPO2 had PO activity after staining (Fig. 2G–2J). The activity of rPPO2-GFP in cell lysates is the same as that of rPPO2 when Cu^2+^ was added during cell transfection (Fig. 2K). Cu^2+^ had no influence on either rPPO2 or rPPO2-GFP expression in S2 cells (Fig. 2L), but was required for PO activation induced by ethanol.

**Figure 3:**
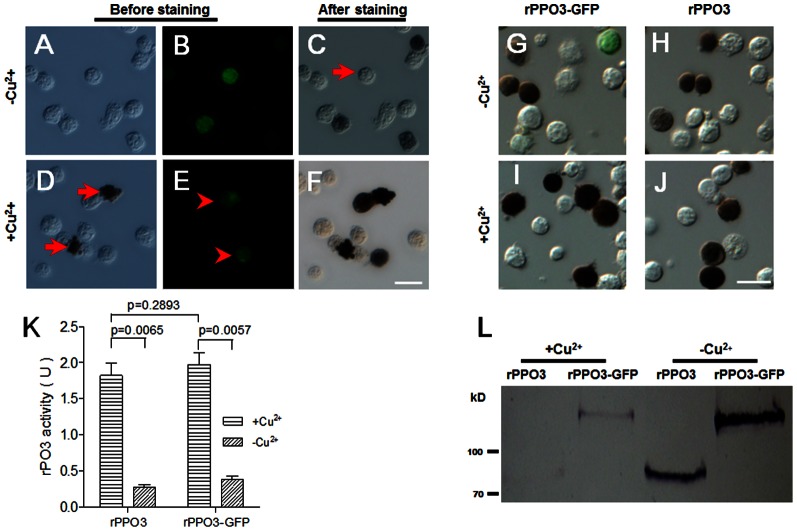
Phenoloxidase in S2 cells over-expressing rPPO3-GFP. DIC and fluorescence images were taken in the absence (A–C; −Cu^2+^) or presence (D–F; +Cu^2+^) of Cu^2+^ during cell transfection. At 48 h, cells with green fluorescence were detected (A, B, D, E), and samples were then stained for PO activity (C, F) as described in Figure 1A–F. When Cu^2+^ was absent, some cells with green fluorescence (B) were stained dark brown (C), which is different from rPPO1-GFP (Figure 1) and rPPO2-GFP (Figure 2). There were also cells with green fluorescence that were not stained for PO activity (C). When Cu^2+^ was present, cells auto-melanized (see arrow in D). The arrowheads point to cells (E) with green fluorescence that were positively stained. (G-J) rPPO3 and rPPO3-GFP were over-expressed in S2 cells in the absence (G, H) or presence (I, J) of Cu^2+^ for 48 h for comparison. In the absence of Cu^2+^, there were still many cells with PO activity (G, H), and many cells were also fluorescent but without PO activity (G). When Cu^2+^ was present during transfection, many cells expressing rPPO3 or rPPO3-GFP were stained dark brown (I, J), and some of these cells were auto-melanized as shown in (D). (K) rPO3 activity assay as shown in Figure 1K. When Cu^2+^ was present, rPPO3 had almost the same enzyme activity as rPPO3-GFP, and these levels were significantly higher than when Cu^2+^ was not added during transfection. Columns represent the mean of individual measurements ± S.E.M (n = 3). Significant differences were calculated using the unpaired *t* test. (L) Western blot showing that rPPO3-GFP and rPPO3 protein expression occurred regardless of the presence or absence of Cu^2+^. However, due to auto-melanization, in the presence of Cu^2+^ some rPPO3 and rPPO3-GFP form large complexes that were not easily separated by SDS-PAGE. Thus, the amounts of rPPO3 and rPPO3-GFP seem lower than those without Cu^2+^ added. Bar: 20 µm.

Similar analyses showed that rPPO3-GFP enzymatic activity was comparable to that of rPPO3. If Cu^2+^ was not added during cell transfection, many cells that express rPPO3-GFP, as indicated by green fluorescence, were stained black (Fig. 3A–3C). However, in the absence of Cu^2+^, some cells stained for PO activity, and some other cells with fluorescence had no PO activity after staining (Fig. 3A–3C). When Cu^2+^ was added during cell transfection, many S2 cells became auto-melanized and had no fluorescence, likely due to fluorescence quenching by melanin (Fig. 3D–3E). However, all cells with visible green fluorescence were stained black after direct addition of dopamine (Fig. 3D–3E). When rPPO3 was compared to rPPO3-GFP, cell staining was similar when Cu^2+^ was not added (Fig. 3G–3H). Some cells with green fluorescence lacked PO activity when rPPO3-GFP was expressed (Fig. 3G), and this is likely due to the presence of newly produced rPPO3-GFP but the absence of sufficient Cu^2+^ to complete the conformation change of the enzyme. But if Cu^2+^ was added, all cells expressing rPPO3 or PPO3-GFP were positively stained for PO activity (Fig. 3I and 3J). Many S2 cells with rPPO3 or rPPO3-GFP expressed were stained black even if Cu^2+^ was not added, which is different from rPPO1, rPPO1-GFP, rPPO2 and rPPO2-GFP. Furthermore, rPPO3 and rPPO3-GFP activity was similar when Cu^2+^ was added (Fig. 3K). However, rPPO3-GFP had significantly lower enzyme activities when Cu^2+^ was not added which is the same as rPPO3. Finally, PPO3 could not be efficiently detected when Cu^2+^ was added, suggesting that this enzyme may form large complexes with other proteins during the progress of auto-melanization, and that these complexes cannot be dissociated during Western blot analyses (Fig. 3L). When phenylthiourea (PTU) was added with Cu^2+^ to inhibit auto-melanization, the protein levels were the same as the group without Cu^2+^ added (data not shown).

The above experiments show that each rPPO-GFP has almost similar properties as the corresponding wild type rPPO, although there might be some differences in their enzymatic activities. The information is summarized in Table 1.

### Co-expression of rPPO1-GFP or rPPO3-GFP with rPPO2 -DsRed

**Figure 4:**
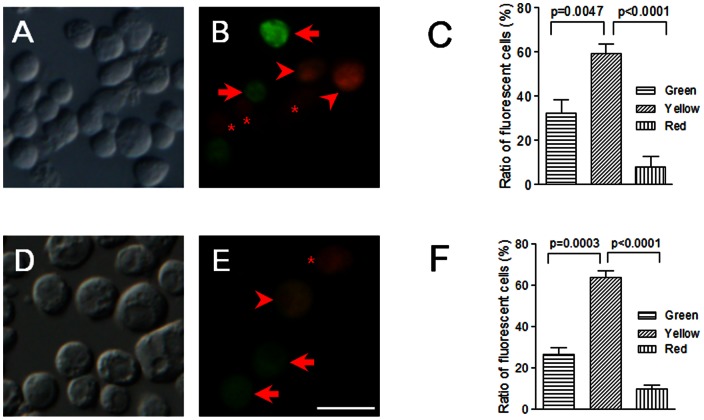
Co-expression of rPPO2-DsRed with either rPPO1-GFP or rPPO3-GFP. Plasmids containing rPPO1-GFP and rPPO2-DsRed (A-B) or rPPO3-GFP and rPPO2-DsRed (D-E) were co-transfected into S2 cells in the absence of Cu^2+^. Cells with green fluorescence (arrows) expressed rPPO1-GFP or rPPO3-GFP, and cells with red fluorescence (asterisks) expressed rPPO2-DsRed. Cells with yellow fluorescence (arrowheads) co-expressed rPPO2-DsRed and either rPPO1-GFP (B) or rPPO3-GFP (E). All images were taken using green and red filters, and were then merged. (C-F) Among PPO positively stained S2 cells, over 60% had yellow fluorescence, approximately 25% had green fluorescence, and less than 10% had red fluorescence (C, F). Obviously, the ratio of cells with yellow fluorescence is significantly higher than that with red or green fluorescence but not all cells were expressing multiple rPPOs at the same time. Columns represent the mean of individual measurements ± S.E.M (n = 4). Significant differences were calculated using the unpaired *t* test program. Bar: 20 µm.

Although there are 2–3 PPO genes in most insects, we do not know whether they can be expressed together in the same cells. Previous work shows that the percentages of positively-stained S2 cells were different if each rPPO was over-expressed [36]. To test it, another plasmid containing rPPO2-DsRed was constructed. rPPO1-GFP/rPPO2-DsRed or rPPO3-GFP/rPPO2-DsRed were co-transfected into S2 cells without Cu^2+^ added. Forty-eight hours later, S2 cells were observed and counted. For each trial, we always observed that there were S2 cells with green fluorescence, red fluorescence, or with green and red fluorescence at the same time (yellow fluorescence after merging; Fig. 4B, 4E). Green fluorescence indicates the expression of rPPO1-GFP or rPPO3-GFP alone. Red fluorescence indicates the expression of rPPO2-DsRed. Yellow fluorescence means that either rPPO1-GFP or rPPO3-GFP was co-expressed with rPPO2-DsRed in the same cells. While the majority of cells expressed both transfected PPOs, statistical evaluation as shown in Fig. 4C and 4F indicates that plasmids containing different rPPO genes are not always over-expressed in the same cells.

### Activation of rPPO-GFP by Serine Protease

**Figure 5:**
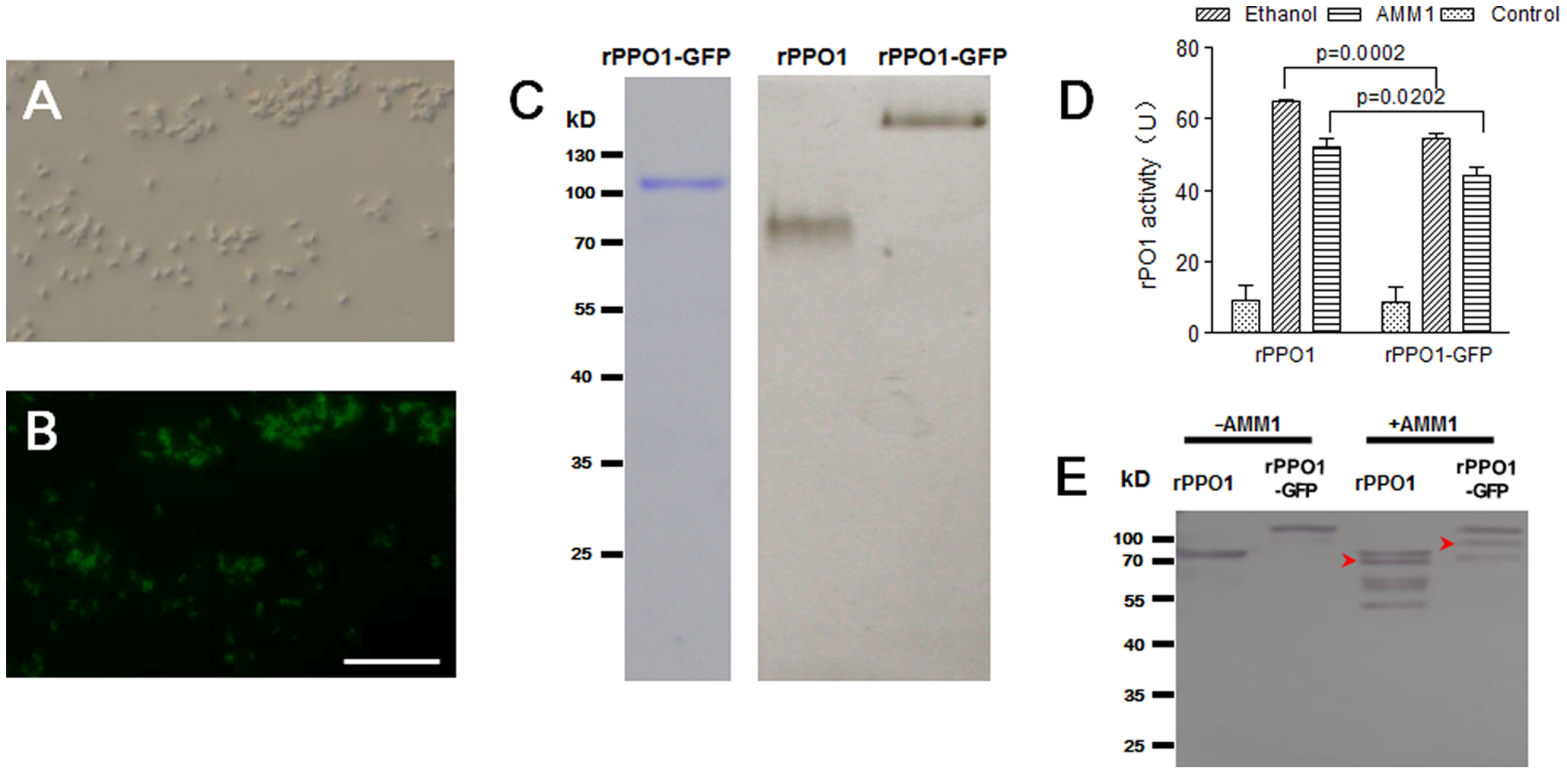
rPPO1-GFP can be activated by serine protease. (A, B) rPPO1-GFP was expressed in *E. coli* cells exhibited green fluorescence (B) as compared with those observed using DIC (A). (C) Purified rPPO1-GFP (0.3 µg) was separated by SDS-PAGE and stained with Coomassie Brilliant Blue, showing that rPPO1-GFP had been purified to homogeneity (left panel in C). rPPO1 (1 µg) and rPPO1-GFP (1 µg) all had enzyme activity in the native gel but their positions were shifted due to the GFP-tag at the C-terminus (right panel in C). (D) Serine proteases activate rPPO1-GFP. A 50 µl system containing purified rPPO1 (3 µg) or rPPO1-GFP (3 µg) and 3 µg AMM1 in 10 mM Tris buffer (pH 8.0) was incubated on ice for 1 h. Ethanol was also used to activate rPPO1 and rPPO1-GFP, and this was done by mixing 3 µg purified protein with an equal volume of 60% ethanol solution and incubating for 5 min. rPPO1 activity is significantly higher than rPO1-GFP when enzymes were activated either by ethanol or AMM1. Columns represent the mean of individual measurements ± S.E.M (n = 6). Significant differences were calculated using the unpaired *t* test. (E) Western blot showing that rPPO1 and rPPO1-GFP were cleaved by serine protease in AMM1 (arrowheads). Bar: 20 µm.

Cells expressing rPPO-GFP or rPPO-DsRed are easily detected using fluorescence microscopes, and the PPO enzyme can be activated by ethanol (Fig. 1K, 2K, 3K). With this knowledge, we sought to determine whether rPPO-GFP can be cleaved and activated by serine proteases, and this was done using rPPO1-GFP purified from transformed *E. coli*. These transfected bacteria cells exhibit green fluoresce, indicating that they express rPPO1-GFP (Fig. 5A and 5B). After purification (Fig. 5C, left panel), rPPO1 and rPPO1-GFP were separated in native gels and then analyzed for PO activity. These blots showed that the purified enzymes contained PO activity (Fig. 5C, right panel). Purified rPPO1 and rPPO1-GFP were activated using ethanol and AMM1 respectively, and they all had enzyme activity (Fig. 5D). rPPO1 and rPPO1-GFP were cleaved by serine proteases, leading to the conversion to rPO1 and rPO1-GFP (Fig. 5E). Therefore, the GFP-tag does not disturb serine protease cleavage of fusion proteins.

### rPPO1-GFP Binds to Bacteria and Fungi

**Figure 6:**
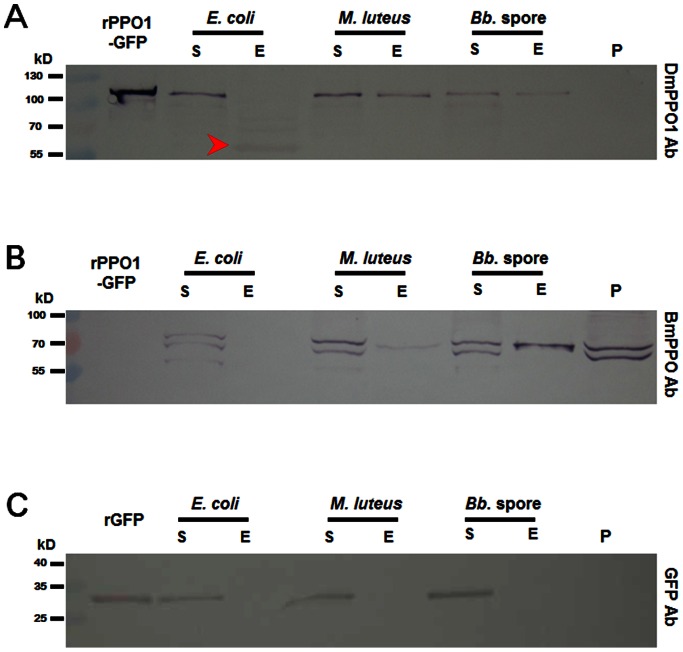
rPPO1-GFP binds to microorganism. Purified rPPO1-GFP (5 µg) or rGFP (5 µg) was mixed with silkworm plasma (5 µl) in a 200 µl system prepared in 50 mM Tris buffer (100 mM NaCl, pH 8.0). Then, different microorganisms were added to the above mixture and incubated at room temperature for 30 min. Proteins bound to microorganisms were eluted using 1×SDS loading buffer and heated at 95°C for 5 min prior to being subjected to Western blot assay. Purified rGFP was used as a control to see whether the binding was due to rGFP (C). Antibodies against *Drosophila* rPPO1 (A; DmPPO1 Ab), *Bombyx* PPO (B; BmPPO Ab) and GFP (C; GFP Ab) were used to detect the corresponding proteins. rPPO1-GFP (A) and silkworm plasma PPO (B) can all bind to *Micrococcus* and *Beauveria bassiana* spores (*Bb.* spore). A smaller band (arrowhead-pointed) was eluted from *E. coli* cells that might be cleaved from rPPO1-GFP (A). The purified rGFP did not bind to microorganisms (C). For each lane, approximately 200 ng purified rPPO1-GFP or rGFP was loaded to indicate their positions. S: supernatant after binding; E: eluted protein; P: silkworm plasma.

*M. sexta* PPO is known to bind to gram-positive bacteria like *Bacillus thuringiensis* and *Staphylococcus aureus* and gram-negative bacteria like *Serratia marcescens* but not *E. coli* [42]. In order to determine whether rPPO-GFP can be used for innate immunity studies, purified rPPO1-GFP was mixed with plasma from silkworm larvae. Approximately 1×10^9^ *E. coli*, or 1 mg dried *Micrococcus*, or 1 mg wet *Beauveria bassiana* spores (*Bb*. Spore) were mixed with 5 µl plasma containing rPPO1-GFP (5 µg) in a 200 µl incubation system. Western blot assays show that rPPO1-GFP is present in the eluted solution when *Micrococcus* and *Beauveria bassiana* spores were used for the binding assay (Fig. 6A). There was also degraded rPPO1-GFP found in the eluted solution when *E. coli* was used for a pull-down. Moreover, when antibody against *Bombyx mori* PPO was used, silkworm PPO was also able to bind to *Micrococcus* and *Beauveria* spores but not to *E. coli* (Fig. 6B). In the silkworm plasma, a smaller band was detected by PPO antibody. However, this band is smaller than rPO1 (data not shown), indicating that it is not activated phenoloxidase. Purified rGFP did not bind to any of the microbes assayed (Fig. 6C). The above results indicate that if there was rPPO1-GFP in larval hemolymph, the fusion protein should be involved in the immune response against various microorganisms.

## Discussion

PPO has many important physiological functions, such as the melanization of invading pathogens and parasites, wound healing and cuticle sclerotization [3]. Insect PPO belongs to type 3 copper-containing proteins that extensively exist in mammals, insects, shrimp, plants and microbes [3]–[7]. The biochemical properties of insect PPOs are conserved. Although we have a good understanding of insect PPO activation, many questions remain [1]. One of them is that PPO is thought to be released through hemocytes rupturing in the hemocoel [3]. However, PPO is produced in the silkworm hindgut and is continuously released into the hindgut lumen for clearance of microbe flora through the melanization of feces [16]. Obviously, PPO released from hindgut cells is different from that released from hemocytes since there is no proof that hindgut cells lyse during release. If we could add a tag like GFP to PPO, it should be easier to determine how PPO is released into the hemolymph or to trace its movement when the insect hosts are wounded or infected.

In this study, GFP was fused at the C-termini of three different *Drosophila* PPOs. In addition, DsRed was fused with *Drosophila* rPPO2 for a co-expression assay. Fluorescence and enzymatic assays showed that when Cu^2+^ was added, S2 cells that had fluorescence also had PO activity when ethanol was used for activation (Fig. 1, 2, 3), and that each rPPO-GFP possessed similar biochemical properties as the corresponding non-GFP tagged rPPO (Table 1). Just as rPPO1, rPPO1-GFP could be activated by serine proteases to show rPO1 activity (Fig. 5D), which indicates that the GFP-tag has no influence on PPO activation. rPO1 activity in S2 cells is lower than that in *E. coli* after expression (Fig. 1 and Fig. 5). The exact reason is unclear. We conclude that rPPO1 expressed in S2 cells in S2 cells may be modified more or less to loss some activity, which needs further studying in the future. The crystal structure of *M. sexta* PPO indicates that PPO1 and PPO2 can form heterodimer in a back-to-back mode [43], [44]. We have no idea whether the heterodimers can be broken during the process of activation after being cleaved by serine proteases. However, GFP fused at the C-terminus did not break down the structure of PPO since each rPPO-GFP in cell lysates had enzyme activity (Fig. 1–3). The tagged GFP can decrease rPO1 activity (Fig. 1K). Therefore, a dimer structure or grafting a different protein at C-terminus may affect the entrance of substrate into the active site pockets of rPO1. However, the tagged GFP at the C-terminus has no obvious effect on rPO2 activity or rPO3 activity (Fig. 2K and 3K). We conclude that there might be some unknown factors in S2 cells that probably affect the activated rPO1-GFP but not rPO2-GFP or rPO3-GFP activities. Obviously, GFP does not interrupt the progress of PPO activation induced either by ethanol or serine protease but it may affect enzyme activities. It is a new different biochemical property among three *Drosophila* PPOs since they can be fused with GFP-tag and still have enzyme activities. When rPPO1-GFP was expressed inside S2 cells, it cannot be induced into secretion into medium with or without *Micrococcus* or LTA *in vitro* (Fig. S2). We believe rPPO-GFP may be helpful to trace PPO released from crystal cells *in vivo* if we could construct a transgenic fly to over-express those proteins in hemocytes.

In the previous work, we found that the transfection efficiency for the three rPPO genes is different [36]. It seems that rPPO3 has the highest transfection efficiency, rPPO1 is medium, and rPPO2 is the lowest. GFP was used to monitored cell transfection efficiency so as to maintain the working conditions as the same each time. When rPPO2-DsRed was co-expressed with rPPO1-GFP or rPPO3-GFP, cells with both green and red fluorescence had rPPO2-DsRed and rPPO1-GFP or rPPO3-GFP co-expressed at the same time. However, the data show that there are always some cells with green or red fluorescence alone, which indicates that there are some S2 cells that are not suitable for specific rPPO expression. In those cells the transfected plasmid might be degraded or protein translation is inhibited by an unknown mechanism. In addition, the S2 cell is a type of hemocyte cell line [45]. Since PPO1 and PPO2 can only be expressed in crystal cells [35], and PPO3 is expressed in lamellocytes [37], this may partly explain different expression of three *Drosophila* PPO genes in S2 cells in this work and a previous paper [36].

*In vivo*, plasma PPO can bind bacteria to initiate immune responses [8], [25], [42]. Here, when rPPO1-GFP was mixed with silkworm plasma for a bacteria or *Beauveria* spores binding assay, rPPO1-GFP was observed to bind to *Micrococcus* cells and fungal spores (Fig. 6A). When antiserum against the silkworm PPO was used, silkworm PPO was clearly observed to bind *Micrococcus* cells and *Beauveria* spores (Fig. 6B). Therefore, rPPO-GFP is involved in the recognition of pathogens. In addition, rPPO1-GFP still has PO activity and induces melanization after being activated and provided substrates. Together, the above work indicates that if rPPO-GFP could be produced *in vivo*, it would facilitate studies on the role of PPO in insect innate immune responses.

## Materials and Methods

### rPPO-GFP and rPPO-DsRed Expression

Three *Drosophila melanogaster* prophenoloxidase (PPO) genes were separately fused with GFP (for PPO1, PPO2 and PPO3) or DsRed (for PPO2 alone) at the C-terminus using overlapping PCR. The primer pairs are listed in Table S1. The PCR products were sub-cloned into pAc5.1/V5-HisB (Invitrogen) under the control of the *Drosophila* actin 5C promoter. Wild type rPPO, rPPO-GFP and rPPO-DsRed were transiently transfected into S2 cells using Effectene™ (Qiagen) as previously described [36]. Copper dichloride (CuCl_2_, 500 µM Sigma), if needed for apo-rPPO or apo-rPPO-GFP to become holo-rPPO or holo-rPPO-GFP, was added to the culture medium during transfection [36]. Cells were usually ready for use at 48 h post transfection.

### rPPO-GFP Detection in S2 Cells

After being seeded on a glass slide, S2 cells with green fluorescence were detected and imaged using a fluorescence microscope (Olympus BX51) with differential interference contrast optics (DIC) and appropriate fluorescence filters. Insect PPO can be cleaved by a specific serine protease for activation [3], [19], [21]. This enzyme can also be activated by some detergents and various kinds of alcohol like ethanol, methanol and 2-propanel [3]. We do not know the mechanism of PPO activation induced by ethanol. However, it is a convenient to detect PPO through ethanol activation for the subsequent staining [36]. PPO inside hemocytes can also be activated by ethanol for staining [14]. Thus, in the same way, S2 cells were stained to detect PO activity using 10 mM dopamine (Sigma) freshly dissolved in 30% ethanol for 30 min [36], by which we performed enzyme activation and staining at the same time. The relationship between fluorescence and PO activities was then compared.

### Enzyme Activity Assay

rPPO-GFP and rPPO were transiently expressed in S2 cells as described for 48 h with or without Cu^2+^ added [36]. S2 cells were collected and suspended in 10 mM Tris buffer (pH 7.4), exposed to three rounds of freezing at −80°C for 2 min, and quickly thawed to room temperature for 4 min. Cells were examined using a microscope to ensure that they had lysed. Some cell lysates, including cell pellets, were used for Western blot assays using each purified rPPO (0.2 µg) as a standard to determine the amount of rPPO or rPPO-GFP expressed in S2 cells. This quantification was performed using ImageJ software (National Institutes of Health). To determine the amount of rPPO3 and rPPO3-GFP expression in S2 cells when Cu^2+^ was added, phenylthiourea (PTU) was added to avoid auto-melanization [36]. Subsequently, the same amounts of each rPPO or rPPO-GFP (1 µg) in S2 cells lysate including cell pellets were mixed with ethanol (30% final concentration) and incubated at room temperature for 5 min. The above mixtures were separately added to 200 µl dopamine (10 mM) and incubated for 9 min at room temperature. The samples were then centrifuged at 10,000 g for 1 min, the supernatants (200 µl) were transferred into microplate wells and the absorbance of the supernatant at 490 nm was measured using the EXPERT 96 microplate reader (Biochrom). One unit of activity was defined as an increase of 0.001 absorbance units/min/µg rPPO.

### Purification of rPPO-GFP after Expression in *Escherichia coli BL21* (DE3)

The *Drosophila* three PPO genes can be expressed in *Escherichia coli (E. coli)* for purifying large amount of rPPO [38]. Although PPO can be expressed inside eukaryotic cells like S2 cells, the production is too low for obtaining large amount of PPO for various biochemical studies. rPPO1-GFP was sub-cloned into pET28a (Invitrogen) for protein expression in *E. coli* at 16°C as described [38]. *E. coli* cells with rPPO1-GFP expressed were observed via microscopy to detect GFP fluorescence before protein purification. As a control, rPPO1 was also expressed in *E. coli* as previously described [38]. rPPO1 and rPPO1-GFP were purified as described but with some modifications [38]. Briefly, bacteria cell pellets were collected after centrifugation and re-suspended in 20 ml Lysis Buffer (10 mM Tris–HCl pH 7.4, 0.2 M NaCl, cells from 250 ml *E. coli* culture). The above suspension was incubated on ice for 30 min, followed by sonication on ice. After that, the cell lysate was centrifuged at 13,800 g for 30 min at 4°C. Two milliliters of 50% Ni-NTA slurry was equilibrated using 20 ml Lysis Buffer in a 10 ml empty column. The supernatant was added to the equilibrated column with the bottom outlet capped and mixed gently. The column was placed at 4°C for 60 min to allow protein binding. The column was then washed using Washing Buffer (10 mM Tris, 0.2 M NaCl, 20 mM Imidazole, pH 7.4) until the absorbance at 280 nm was not changed. rPPO1 or rPPO1-GFP was eluted with Elution Buffer (10 mM Tris, 0.2 M NaCl, 100 mM Imidazole, pH 7.4). For each elution fraction, 500 µl solution was collected. The purity of rPPO1 or rPPO1-GFP in each fraction was determined by SDS-PAGE. The fractions containing rPPO1 or rPPO1-GFP with high purity were combined and then the purified rPPO1 or rPPO1-GFP was ultrafiltrated using changing buffer (10 mM Tris, pH 7.4) at 8,000 g at 4°C for 5 times. Next the concentrations of purified proteins were determined. For long time preservation at −80°C, 50% glycerol (final concentration) was added and the samples aliquoted. The above purified rPPO1 and rPPO1-GFP then must be added to Cu^2+^ to achieve a status that can be activated by ethanol or serine proteases [38].

### Activation of Purified rPPO1 and rPPO1-GFP by Serine Protease

A protein fraction containing serine proteases from *Drosophila melanogaster* initially called AMM1 [46], was prepared as described [47], [48]. Purified rPPO1 (3 µg) and rPPO1-GFP (3 µg) were then incubated with 3 µg AMM1 in a 50 µl system prepared in 10 mM Tris buffer (pH 8.0) on ice for 1 h. As a comparison, rPPO1 and rPPO1-GFP were also activated by mixing 3 µg purified protein with an equal volume of 60% ethanol solution and incubating for 5 min. The amount equal to original rPPO1 (0.25 µg) was then removed and mixed with 200 µl dopamine (10 mM) dissolved in 10 mM Tris-buffer (pH 7.5), and the absorbance at 490 nm was continuously monitored using an EXPERT 96 microplate reader. To identify rPPO1 and rPPO1-GFP cleavage by AMM1, an amount equal to 0.5 µg protein was used in a Western blot assay.

### Microorganisms Preparation and Binding Assay

*E. coli* was prepared as described [16]. *Micrococcus luteus* (Sigma) was suspended in sterile 0.85% NaCl. *Beauveria bassiana* spores were prepared as described [49] and suspended in sterile 0.85% NaCl. *Micrococcus* and *bassiana* spores (wet weight) were re-suspended to make a concentration 10 mg/ml.

Insect plasma PPO forms a large complex with other plasma proteins to bind various microorganisms [42]. In order to identify whether rPPO tagged with GFP has such an innate immunity function, 5 µg purified rPPO1-GFP was mixed with 5 µl plasma from naïve *Bombyx* larvae suspended in a 200 µl system prepared in binding buffer (50 mM Tris buffer, 100 mM NaCl, pH 8.0). To inhibit the auto-melanization of plasma, 5 µl saturated phenylthiourea (PTU) was added and incubated on ice for 30 min. Approximately 1×10^9^ *E. coli* cells or 1 mg dried *Micrococcus* or 1 mg *Beauveria bassiana* spores were suspended in the above mixture and incubated for 30 min at room temperature as described with modification [42]. The mixtures were centrifuged at 13,000 g for 5 min to pellet the microorganisms and the supernatant was removed. After vortexing and washing with 0.2 ml binding buffer three times, the pellets were suspended in 1×SDS (200 µl) loading buffer and heated to 95°C for 5 min. The supernatants containing eluted proteins were used in a Western blot assay. To replace rPPO1-GFP, purified rGFP was also used for binding assay as described above.

### LC-MS/MS

rPPO1-GFP expressed in S2 cells was identified using a LC-MS/MS assay to ensure that there was no problem with the expression system for different fusion proteins. When rPPO1-GFP was over-expressed in S2 cells for 48 h, cells were then collected and lysed. Approximately 15 µg total proteins were loaded for a denatured SDS-PAGE assay followed by Coomassie Brilliant Blue R-250 staining. Based on Western blot detection, the gel band containing rPPO1-GFP was excised for LC-MS/MS assay as described [16]. The *Drosophila* protein sequence database used in this analysis was downloaded from UniProt (http://www.uniprot.org/) using the keyword “*Drosophila melanogaster*” plus green fluorescent protein (GenBank: ADQ43426.1).

### S2 Cells Immune Response

rPPO1-GFP was over-expressed in S2 cells for 48 h with 0.5 mM CuCl_2_ added in culture medium as described [36]. Then 6 µg Lipoteichoic acid from *Bacillus subtilis* (LTA) (L3265, Sigma) or 60 µg dried *Micrococcus luteus* cells was added to S2 cells, respectively. The above cells were collected after 4.5 h incubation. S2 cells were centrifuged and lysed. Approximately 6 µg total proteins or 15 µl medium were loaded per lane for a Western blot assay using a polyclonal antibody against *Drosophila* rPPO1.

### Western Blot Assays

Western blot was performed to detect rPPO or rPPO-GFP expressed in S2 cells as previously described [36]. Rabbit anti-His-tag polyclonal antibody (ABCAM; 1∶3, 000) was used to detect each rPPO and rPPO-GFP. Antibodies against *Drosophila* rPPO1 or against silkworm PPO [50] do not cross react. Therefore, antibodies against *Drosophila* rPPO1 (1∶3, 000), *Bombyx* PPO (1∶5, 000) and rGFP (1∶2, 000) were used as the primary antibodies for detecting the corresponding protein in the microorganism binding assay. Goat anti-rabbit IgG conjugated with alkaline phosphatase (AP, Chemicon) (1∶5, 000) was used as the secondary antibody. In order to detect whether rPPO1-GFP can be released into medium from S2 cells, proteins were detected by chemiluminescence catalyzed by a horseradish peroxidase (HRP) conjugated secondary antibody using the Pierce ECL Western Blotting Substrate (32106; Thermo).

## Supporting Information

Figure S1 **Separation of S2 cell lysate proteins for LC-MS/MS assay.** S2 cells transfected with blank vector (as control) or rPPO1-GFP were cultured for 48 h, and then cells were collected and lysed. (A) Protein samples of S2 the control cell lysate (left lane) and the one containing over-expressed rPPO1-GFP (right lane) were separated on SDS-PAGE for Coomassie Brilliant Blue staining. Approximately 15 µg total protein were loaded. (B) Western blot using polyclonal antiserum against rPPO1 to locate rPPO1-GFP position. Another gel was made as shown in (A) for western blot assay. After comparison, the framed area (right lane in A) was excised for LC-MS/MS assay.
(TIF)

Figure S2 **rPPO1-GFP is not released into culture medium.** rPPO1-GFP was over-expressed in S2 cells for 48 h, and then LTA and *Micrococcus* were added to those S2 cells respectively for 4.5 h. S2 cells (6 µg) and culture medium (15 µl) were prepared for Western blot assay using polyclonal antibody against *Drosophila* rPPO1. rPPO1-GFP is not released into culture medium. M: cell culture medium; C: cells with rPPO1-GFP over-expressed.
(TIF)

Table S1 **List of primers used for fusing each PPO with GFP or DsRed.**
(XLS)

Table S2 **List of proteins identified by LC-MS/MS.** The framed gel containing rPPO1-GFP as shown in Figure S1 was excised for LC-MS/MS assay. All identified proteins are listed. rPPO1 (GenBank: AAF57775.1) and GFP (GenBank: ADQ43426.1) were labeled in green. Fusion rPPO1-GFP was expressed in S2 cells.
(XLS)

## References

[1] Kanost MR, Gorman MJ (2008) Phenoloxidases in insect immunity. In: Beckage NE, editor. Insect immunology. San Diego: Academic Press. 69–96.

[2] Ashida M (1971) Purification and characterization of pre-phenoloxidase from hemolymph of the silkworm *Bombyx mori* . Arch Biochem Biophys 144: 749–762. 4328165 10.1016/0003-9861(71)90383-3

[3] Ashida M, Brey P (1998) Recent advances on the research of the insect prophenoloxidase cascade. In: Brey P, Hultmark D, editors. Molecular mechanisms of immune responses in insects. London: Chapman & Hall. 135–172.

[4] Fairhead M, Thöny-Meyer L (2012) Bacterial tyrosinases: old enzymes with new relevance to biotechnology. N Biotechnol 29: 183–191. 21664502 10.1016/j.nbt.2011.05.007

[5] Olivares C, Solano F (2009) New insights into the active site structure and catalytic mechanism of tyrosinase and its related proteins. Pigment Cell Melanoma Res 22: 750–760. 19735457 10.1111/j.1755-148X.2009.00636.x

[6] Mayer AM (2006) Polyphenol oxidases in plants and fungi: Going places? A review. Phytochemistry 67: 2318–2331. 16973188 10.1016/j.phytochem.2006.08.006

[7] Phonpala Y, Benjakul S, Visessanguan W, Eun J-B (2009) Sulfur-containing compounds heated under alkaline condition: antibrowning, antioxidative activities, and their effect on quality of Shrimp during Iced Storage. J Food Sci 74: S240–S247. 19723229 10.1111/j.1750-3841.2009.01189.x

[8] Hillyer JF, Christensen BM (2005) Mosquito phenoloxidase anddefensin colocalize in melanization innate immune responses. J Histochem Cytochem 53: 689–698. 15928318 10.1369/jhc.4A6564.2005

[9] Zou Z, Shin SW, Alvarez KS, Bian G, Kokoza V, et al (2008) Mosquito RUNX4 in the immune regulation of PPO gene expression and its effect on avian malaria parasite infection. Proc Natl Acad Sci U S A 105: 18454–18459. 19011100 10.1073/pnas.0804658105 PMC2587535

[10] Zou Z, Shin SW, Alvarez KS, Kokoza V, Raikhel AS (2010) Distinct melanization pathways in the mosquito *Aedes aegypti* . Immunity 32: 41–53. 20152169 10.1016/j.immuni.2009.11.011

[11] Bidla G, Dushay MS, Theopold U (2007) Crystal cell rupture after injury in *Drosophila* requires the JNK pathway, small GTPases and the TNF homolog Eiger. J Cell Sci 120: 1209–1215. 17356067 10.1242/jcs.03420

[12] Bidla G, Hauling T, Dushay MS, Theopold U (2009) Activation of insect phenoloxidase after injury: endogenous versus foreign elicitors. J Innate Immun 1: 301–308. 20375588 10.1159/000168009

[13] Strand MR (2008) The insect cellular immune response. Insect Science 15: 1–14.

[14] Ling E, Shirai K, Kanehatsu R, Kiguchi K (2005) Reexamination of phenoloxidase in larval circulating hemocytes of the silkworm, *Bombyx mori* . Tissue Cell 37: 101–107. 15748736 10.1016/j.tice.2004.10.007

[15] Ling E, Yu XQ (2005) Prophenoloxidase binds to the surface of hemocytes and is involved in hemocyte melanization in *Manduca sexta* . Insect Biochem Mol Biol 35: 1356–1366. 16291091 10.1016/j.ibmb.2005.08.007

[16] Shao Q, Yang B, Xu Q, Li X, Lu Z, et al (2012) Hindgut innate immunity and regulation of fecal microbiota through melanization in insects. J Biol Chem 287: 14270–14279. 22375003 10.1074/jbc.M112.354548 PMC3340165

[17] Diao Y, Lu A, Yang B, Hu W, Peng Q, et al (2012) Existence of prophenoloxidase in wing discs: a source of plasma prophenoloxidase in the silkworm, *Bombyx mori* . PLoS ONE 7: e41416. 22848488 10.1371/journal.pone.0041416 PMC3405132

[18] Dittmer NT, Hiromasa Y, Tomich JM, Lu N, Beeman RW, et al (2011) Proteomic and Transcriptomic analyses of rigid and membranous cuticles and epidermis from the elytra and hindwings of the Red Flour Beetle, *Tribolium castaneum* . J Proteome Res 11: 269–278. 22087475 10.1021/pr2009803

[19] Kanost MR, Jiang H, Yu XQ (2004) Innate immune responses of a lepidopteran insect, *Manduca sexta* . Immunol Rev 198: 97–105. 15199957 10.1111/j.0105-2896.2004.0121.x

[20] Cerenius L, Lee BL, Söderhäll K (2008) The proPO-system: pros and cons for its role in invertebrate immunity. Trends Immunol 29: 263–271. 18457993 10.1016/j.it.2008.02.009

[21] Jiang H, Vilcinskas A, Kanost MR (2011) Immunity in lepidopteran insects. In: Söderhäll K, editor. Invertebrate Immunity: Springer US. 181–204. 10.1007/978-1-4419-8059-5_10 PMC9284565 21528699

[22] Zou Z, Picheng Z, Weng H, Mita K, Jiang H (2009) A comparative analysis of serpin genes in the silkworm genome. Genomics 93: 367–375. 19150649 10.1016/j.ygeno.2008.12.010 PMC2772820

[23] Zou Z, Jiang H (2005) *Manduca sexta*serpin-6 regulates immune serine proteinases PAP-3 and HP8: cDNA cloning, protein expression, inhibition kinetics, and function elucidation. J Biol Chem 280: 14341–14348. 15691825 10.1074/jbc.M500570200 PMC2047605

[24] Yu X-Q, Jiang H, Wang Y, Kanost MR (2003) Nonproteolytic serine proteinase homologs are involved in prophenoloxidase activation in the tobacco hornworm, *Manduca sexta* . Insect Biochem Mol Biol 33: 197–208. 12535678 10.1016/s0965-1748(02)00191-1

[25] Hillyer JF, Schmidt SL, Christensen BM (2003) Hemocyte-mediated phagocytosis and melanization in the mosquito *Armigeres subalbatus* following immune challenge by bacteria. Cell Tissue Res 313: 117–127. 12838409 10.1007/s00441-003-0744-y

[26] Lai S-C, Chen C-C, Hou RF (2002) Immunolocalization of prophenoloxidase in the process of wound healing in the mosquito *Armigeres subalbatus (Diptera: Culicidae)* . J Med Entomol 39: 266–274. 11931025 10.1603/0022-2585-39.2.266

[27] Christensen BM, Li J, Chen C-C, Nappi AJ (2005) Melanization immune responses in mosquito vectors. Trends Parasitol 21: 192–199. 15780842 10.1016/j.pt.2005.02.007

[28] Beerntsen BT, James AA, Christensen BM (2000) Genetics of mosquito vector competence. Microbiol Mol Biol Rev 64: 115–137. 10704476 10.1128/mmbr.64.1.115-137.2000 PMC98988

[29] Collins F, Sakai R, Vernick K, Paskewitz S, Seeley D, et al (1986) Genetic selection of a Plasmodium-refractory strain of the malaria vector *Anopheles gambiae* . Science 234: 607–610. 3532325 10.1126/science.3532325

[30] Wang Y, Hao H, Qiu ZW, Xu WY, Zhang J, et al (2009) Involvement of prophenoloxidases in the suppression of Plasmodium yoelii development by *Anopheles dirus* . Exp Parasitol 123: 6–10. 19540233 10.1016/j.exppara.2009.05.017

[31] Coggins SA, Estévez-Lao TY, Hillyer JF (2012) Increased survivorship following bacterial infection by the mosquito *Aedes aegypti* as compared to *Anopheles gambiae* correlates with increased transcriptional induction of antimicrobial peptides. Dev Comp Immunol 37: 390–401. 22326457 10.1016/j.dci.2012.01.005

[32] Tang H (2009) Regulation and function of the melanization reaction in *Drosophila* . Fly (Austin) 3: 105–111. 19164947 10.4161/fly.3.1.7747

[33] Kan H, Kim C-H, Kwon H-M, Park J-W, Roh K-B, et al (2008) Molecular control of phenoloxidase-induced melanin synthesis in an insect. J Biol Chem 283: 25316–25323. 18628205 10.1074/jbc.M804364200

[34] Eleftherianos I, Revenis C (2011) Role and importance of phenoloxidase in insect hemostasis. J Innate Immun 3: 28–33. 21051882 10.1159/000321931

[35] Rizki RM, Rizki TM (1984) Selective destruction of a host blood cell type by a parasitoid wasp. Proc Natl Acad Sci U S A 81: 6154–6158. 6435126 10.1073/pnas.81.19.6154 PMC391878

[36] Liu F, Chen Y, Yang B, Wang J, Peng Q, et al (2012) *Drosophila melanogaster* prophenoloxidases respond inconsistently to Cu^2+^ and have different activity in vitro. Dev Comp Immunol 36: 619–628. 22178920 10.1016/j.dci.2011.12.001

[37] Irving P, Troxler L, Heuer TS, Belvin M, Kopczynski C, et al (2001) A genome-wide analysis of immune responses in *Drosophila* . Proc Natl Acad Sci U S A 98: 15119–15124. 11742098 10.1073/pnas.261573998 PMC64993

[38] Li X, Ma M, Liu F, Chen Y, Lu A, et al (2012) Properties of *Drosophila melanogaster* prophenoloxidases expressed in *Escherichia coli.* . Dev Comp Immunol 36: 648–656. 22120533 10.1016/j.dci.2011.11.005

[39] Nam HJ, Jang IH, Asano T, Lee WJ (2008) Involvement of pro-phenoloxidase 3 in lamellocyte-mediated spontaneous melanization in *Drosophila* . Mol Cells 26: 606–610. 18852525

[40] Chen Y, Liu F, Yang B, Lu A, Wang S, et al (2012) Specific amino acids affecting *Drosophila melanogaster* prophenoloxidase activity in vitro. Dev Comp Immunol 38: 88–97. 22579944 10.1016/j.dci.2012.04.007

[41] Shrestha S, Stanley D, Kim Y (2011) PGE2 induces oenocytoid cell lysis via a G protein-coupled receptor in the beet armyworm, *Spodoptera exigua* . J Insect Physiol 57: 1568–1576. 21867708 10.1016/j.jinsphys.2011.08.010

[42] Ragan EJ (2008) Immune-related protein complexes and serpin-1 isoforms in *Manduca sexta* plasma. Kansas State University (Dissertation).

[43] Li Y, Wang Y, Jiang H, Deng J (2009) Crystal structure of *Manduca sexta* prophenoloxidase provides insights into the mechanism of type 3 copper enzymes. Proc Natl Acad Sci U S A 106: 17002–17006. 19805072 10.1073/pnas.0906095106 PMC2761362

[44] Jiang H, Wang Y, Ma C, Kanost MR (1997) Subunit composition of pro-phenol oxidase from *Manduca sexta*: molecular cloning of subunit ProPO-P1. Insect Biochem Mol Biol 27: 835–850. 9474780 10.1016/s0965-1748(97)00066-0

[45] Schneider I (1972) Cell lines derived from late embryonic stages of *Drosophila melanogaster* . J Embryol Exp Morphol 27: 353–365. 4625067

[46] Seybold W, Meltzer P, Mitchell H (1975) Phenol oxidase activation in *Drosophila*: A cascade of reactions. Biochem Genet 13: 85–108. 806288 10.1007/BF00486009

[47] Asada N, Fukumitsu T, Fujimoto K, Masuda K (1993) Activation of prophenoloxidase with 2-propanol and other organic compounds in *Drosophila melanogaster* . Insect Biochem Mol Biol 23: 515–520. 8508190 10.1016/0965-1748(93)90060-6

[48] Fujimoto K, Masuda K, Asada N, Ohnishi E (1993) Purification and characterization of prophenoloxidases from pupae of *Drosophila melanogaster* . J Biochem 113: 285–291. 8486601 10.1093/oxfordjournals.jbchem.a124040

[49] Shang Y, Duan Z, Huang W, Gao Q, Wang C (2012) Improving UV resistance and virulence of *Beauveria bassiana* by genetic engineering with an exogenous tyrosinase gene. J Invertebr Pathol 109: 105–109. 22024554 10.1016/j.jip.2011.10.004

[50] Asano T, Takebuchi K (2009) Identification of the gene encoding pro-phenoloxidase A(3) in the fruitfly, *Drosophila melanogaster* . Insect Mol Biol 18: 223–232. 19141111 10.1111/j.1365-2583.2008.00858.x

