# Supplementary figures and images for: Activity of Fusion Prophenoloxidase-GFP and Its Potential Applications for Innate Immunity Study
Source: PLoS One. 2013 May 23;8(5):e64106. doi: 10.1371/journal.pone.0064106 (PMC3662757; doi:10.1371/journal.pone.0064106)

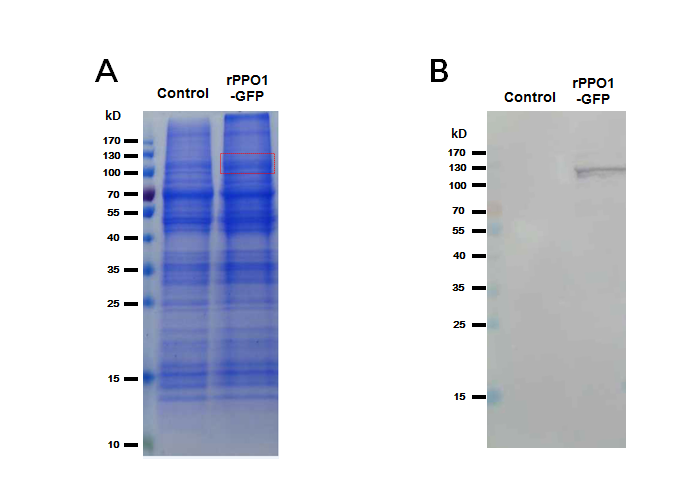

Supplement: Figure S1 — Separation of S2 cell lysate proteins for LC-MS/MS assay. S2 cells transfected with blank vector (as control) or rPPO1-GFP were cultured for 48 h, and then cells were collected and lysed. (A) Protein samples of S2 the control cell lysate (left lane) and the one containing over-expressed rPPO1-GFP (right lane) were separated on SDS-PAGE for Coomassie Brilliant Blue staining. Approximately 15 µg total protein were loaded. (B) Western blot using polyclonal antiserum against rPPO1 to locate rPPO1-GFP position. Another gel was made as shown in (A) for western blot assay. After comparison, the framed area (right lane in A) was excised for LC-MS/MS assay. (TIF) [file pone.0064106.s001.tif]

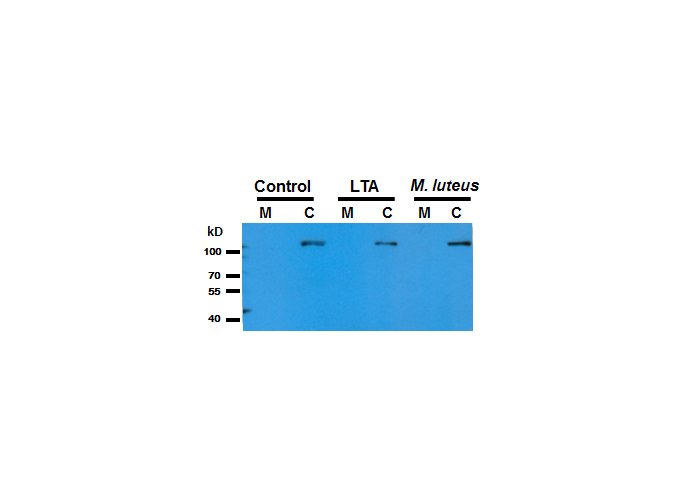

Supplement: Figure S2 — rPPO1-GFP is not released into culture medium. rPPO1-GFP was over-expressed in S2 cells for 48 h, and then LTA and Micrococcus were added to those S2 cells respectively for 4.5 h. S2 cells (6 µg) and culture medium (15 µl) were prepared for Western blot assay using polyclonal antibody against Drosophila rPPO1. rPPO1-GFP is not released into culture medium. M: cell culture medium; C: cells with rPPO1-GFP over-expressed. (TIF) [file pone.0064106.s002.tif]
